# Behavioural indicators of post-release survival in a deep-sea skate

**DOI:** 10.1098/rspb.2025.1345

**Published:** 2025-08-13

**Authors:** Colette Appert, Barrett Wolfe, Sean Tracey, Cara Masere, Simon J. Wotherspoon, Jaimie Cleeland

**Affiliations:** ^1^ Department of Fisheries and Aquaculture, University of Tasmania Institute for Marine and Antarctic Studies, Taroona, Tasmania 7053, Australia; ^2^ Australian Antarctic Division, Kingston, Tasmania 7050, Australia

**Keywords:** mortality, bycatch, elasmobranch, PSAT, telemetry, fisheries

## Abstract

Deep-sea skates are among the most frequently bycaught species in Southern Ocean demersal fisheries. They face heightened susceptibility to fishing pressure due to their life-history characteristics. In longline fisheries targeting Patagonian toothfish, skates caught in good condition are released; however, their post-release survival remains uncertain but is expected to be low, given the extreme capture depths (>1000 m). Post-release survival rates are essential to determine an acceptable fishery mortality for sustainable management. During a 2023 Kerguelen Plateau fishing voyage, 24 satellite tags were deployed on *Bathyraja irrasa* >1056 mm in total length caught at 1200–1600 m depth, for 30 day investigations into post-release survival. Vertical migrations within depth time series indicated that at least six skates survived. Hidden Markov models applied to tag mobility data and summary values of other activity metrics, in the context of topography and currents, were used to determine the fate of skates with no detectable vertical movements, revealing a 26% (95% CI 13–46%) survival rate. The probability of survival decreased with capture depth. Surviving skates underwent extensive non-diel vertical movements. The survival rate is lower than that of other deep-sea skate species with estimated survival rates; which prompts a review of skate bycatch management strategies in deep-set demersal fisheries with high release rates.

## 1. Background

Over one-third of elasmobranch (sharks and rays) species are threatened with extinction, mainly due to fisheries-induced mortality [1,2]. They are generally not the main target of fisheries, but bycatch, which can be poorly recorded and identified [3]. While many elasmobranch studies focus on the population declines of sharks, less attention has been given to rajids (skates) [2,4,5]. Substantial population declines and the extinction of some skate species have gone unnoticed due in part to multi-species management strategies [6]. As shallower fish stocks decline worldwide, fisheries have expanded to deeper dwelling species [7–9], reducing deep refuge for skates [10,11]. Moreover, deep-sea skates are particularly at risk because of their life-history traits. Their slow growth and maturation provide little population resilience when faced with overfishing [12]. Spatial management of fisheries and the creation of ‘no-take’ marine protected areas can reduce fishing pressure [13,14]. Various fisheries management groups and governments have also encouraged or enforced the release of live sharks and rays to reduce fishing mortality [15].

Skates, along with grenadiers (*Macrourus* spp.), represent the greatest biomass of bycatch in Southern Ocean Patagonian and Antarctic toothfish (*Dissostichus* spp.) fisheries. In the Southern Ocean, at least nine species, from two genera, have been described [16]. On the Kerguelen Plateau around Heard Island and McDonald Islands (HIMI), *Bathyraja irrasa* [17], are the most commonly caught species by longline [18]. *B. irrasa* has been listed as vulnerable on the IUCN Red List of Threatened Species following indications of population decline due to its slow maturation and high overlap in distribution with fishing pressure [19,20]. Based on mark-recapture data, they have relatively small home ranges, staying on average within 10 km radii but sometimes travelling over 70 km, and are caught throughout the depth range of the fishery (500−2000 m) [18,21]. They are dependent on the sea floor as their diet is mainly benthic, but their movement patterns are generally unknown [22]. Although there is high uncertainty in stock assessments of the species [23], biomass estimates based on shallow (*<*1000 m) trawling have shown an overall decline since 2019 [24]. The total allowable catch (TAC) for all combined skate species has been fixed at 120 tonnes in HIMI since the late 1990s, but only retained skates are counted towards this TAC since released skates are considered alive [25]. Under the Commission for the Conservation of Antarctic Marine Living Resources (CCAMLR), which manages Antarctic fisheries, ‘any bycatch of shark [...], shall, as far as possible, be released alive’ [26]. As a result, over 90% of *B. irrasa* caught are released in the HIMI fishery, however, their fate is unknown, so the effectiveness of this policy in reducing fishery mortality is uncertain [27].

Encouraging release can effectively reduce fisheries mortality only if skates are landed on the vessel in good condition and post-release survival is high. Fishery mortality is composed of: (i) at-vessel mortality, skates that are observed dead on the vessel and (ii) post-release mortality, skates that die after being released, as a consequence of capture [28]. Post-release mortality is often unaccounted for as it is cryptic. Elasmobranchs can be more or less resilient to capture depending on their physiological and ecological attributes [29]. Pelagic, fast-swimming obligate ram ventilators with high metabolism, for example, are more susceptible to capture stress than more sedentary buccal-pumping species with low metabolism due to their high oxygen requirements and inability to ventilate when stationary [30]. Sudden temperature changes in excess of 9°C can also cause severe metabolic stress [31]. In this respect, skates are physiologically well equipped to sustain the stress of capture at high latitudes, where temperature gradients between capture depths and the surface are minimal [31,32]. However, being hauled from extreme depths may exceed the resilience of skates to capture, potentially leading to low post-release survival [33]. Post-release survival rates are currently unknown for *B. irrasa* in the Patagonian toothfish longline fishery, and as such are not incorporated in total fishery mortality estimates, limiting confidence in the sustainability of current bycatch management.

While assessing post-release survival is challenging even in the best conditions, it is extremely difficult in a remote, deep-sea fishery. Estimated survival can be quantified through condition assessments upon hauling, captive observations, or tagging and release (conventional mark-recapture or satellite tagging), and only very few studies have focused on deep-sea benthic fishes, particularly skates [28,34]. Very low recapture rates for *B. irrasa* in both the French and Australian longline toothfish fisheries on the Kerguelen Plateau have hindered mortality estimation, but point towards low post-release survival for the species [21,32,35]. Captive observations omit the risk of predation and may underestimate long-term effects of capture [33,34]. While vitality assessments can be used as a proxy for likelihood of survival [36], they only provide a prognosis of survival. Condition assessments rely heavily on knowing vital signs and understanding stress responses of an animal to predict its survival after release, extending beyond just observable mortality on the vessel. Aside from a single observational study using remote underwater vehicle footage [37], most deep-sea skates have only been studied through the lens of fishery-dependent research due to the difficulty of accessing these species. Therefore, knowledge of normal physiological baselines and behaviours, beyond bio-metrics is limited for deep-sea skates because most observations occur after the fish have already experienced stress from capture. Pop-up satellite archival tags (PSATs) enable remote assessment of an animal’s condition after it has returned to its habitat. They account for risks of predation, and provide an opportunity to indirectly observe normal behaviour during and after recovery. This study aimed to estimate the post-release survival rates of *B. irrasa* in the HIMI longline Patagonian toothfish fishery using PSATs.

## 2. Methods

### (a) Skate capture, tagging and release

All field work was conducted on a bunker-style longliner (F/V*Cape Arkona*) during a Patagonian toothfish, *Dissostichus eleginoides* [38], fishing voyage to Heard Island in the Southern Ocean, from March to June 2023. The vessel was equipped with automatic systems for baiting, hauling and stripping hooks. Unlike some other vessels in the fishery that use a moonpool for hauling, this vessel uses a side opening for line retrieval, where the catch is initially handled. *B. irrasa*, Kerguelen sandpaper skates, (*n* = 24) were randomly selected from fish caught during standard commercial demersal longlining operations and tagged with PSATs (miniPAT, Wildlife Computers). Longlines consisted of approximately 16 000 m of weighted mainline with approximately 12 000 straight shank hooks (15/0) on 40 cm secondary lines baited with mackerel (*Scomber scombrus*).

During hauling, the vessel speed was approximately 1.5 knots, with hauling speeds approximately 1.6−1.8 ms^−1^; initially slower and accelerating towards the end of the line and as the line section gets shallower. Soak times for the lines were 9−34 h (mean 24 ± 8 h, s.d.) and set depths 1250−1550 m (mean 1400 ± 110 m, s.d.). Fishing occurred continuously throughout the day and night. In this fishery, observers must monitor at least 45% of every line hauled to record catch composition [39].

During normal fishing operations, when a skate is observed during hauling, the autoline is stopped to cut the secondary line to the skate before it reaches the roller. The roller is a rotating cylinder that guides and reduces friction on the ascending line (e.g. MA HV 200, Mustad Autoline); there is usually an automatic de-hooker (e.g. MA HC 100 Standard HookCleaner, Mustad Autoline) right after the cylinder that could seriously injure skates if it were not cut off the main line before. The skate is then lifted aboard, assessed and de-hooked before being retained or released [40]. Releasable skates were randomly handed over to the research team and placed on a table and their eyes and spiracles covered with a soaked towel for de-hooking, condition assessment, length measurement, sexing, blood sampling and tagging with a PSAT and two T-bar tags before being released back into the water. Skates were tagged with T-bar tags, flexible, external tags with a unique identifier as part of a long-term mark-recapture monitoring programme in the fishery. Time out of water was 5 min on average and did not exceed 9 min. Skates were released from the hauling room, 2−4 m a.s.l., as per normal longline fishing operations. The condition assessment used in this study follows the CCAMLR-endorsed condition assessment for skates [36]. A skate in good condition can have red or pink gills, minimal bruising and parasitism and was hooked in the jaw cartilage or soft tissue surrounding the jaw with minimal to moderate tissue damage. Skates with white gills, protruding organs, hooked in the gills or any internal organ or with a broken or ripped out jaw have a severe prognosis and must be retained. All skates that did not meet the release conditions or were smaller than 1056 mm total length (approximately 8 kg) were excluded. The minimum size was set based on the length–weight relationship for this species, to mitigate against drag or positive buoyancy from the tag, which could disproportionately affect the swimming efficiency or behaviour of smaller individuals [41]. One tag, hereafter named the control tag, was deployed attached to a euthanized skate that was landed with a severe prognosis.

MiniPATs were attached to the tail following Le Port *et al*. [42] with an added silicone pad on the ventral side of the tail to avoid ulceration, as in [43] (electronic supplementary material, S1).

### (b) Tag programming and data analysis

MiniPATs (*n* = 24) were programmed to record and transmit depth and acceleration metrics. Two pilot tags were programmed to pop up after 5 days, while the remaining 22 were programmed to pop up after 30 days. A 30 day deployment was chosen as a compromise between the high-data resolution necessary to detect potential mortality, given the lack of knowledge about behavioural patterns and ensuring the tag could transmit a sufficient proportion of messages to the Argos satellite system once surfaced. The tags deployed for 30 days transmitted depth time series and a ‘high activity count’ metric, based on acceleration measurements, at 150 s intervals (see [44] for further details on parameters). The miniPAT depth sensor has a resolution of 0.5 m and accuracy that decreases with depth ( ±1% of reading). Therefore, accuracy at 1700 m is ±17 m. Additionally, the satellite-transmitted time series depth values are binned by dividing the depth range encountered during a 2 h summary period into 16 steps. If the skate had not moved in 2 h the added imprecision will be low (*<*0.1 m) but if the depth range is 160 m, each depth record is a 10 m bin. The mean of the tag mobility metric (mean standard deviation of the sum of acceleration in each axis Ax, Ay and Az over a 3 s window recorded every second) and percentage of time ‘upright’ were calculated over 2 h periods. Tag mobility is a unit-less metric with recorded values 5−63. The ‘upright’ threshold was set at −0.75 g; based on the only other known use of this miniPAT metric, which was on Pacific halibut [45]. Every second, the tag was either upright (*>*−0.75 g) or tilted (*<*−0.75 g). The percentage of time upright within each 2 h period was transmitted. Data and code are available on the Institute for Marine and Antarctic Studies data repository [46]. For tags that were opportunistically recovered (*n* = 2), a complete 1 Hz dataset for depth, temperature, *z*-axis acceleration and tag mobility time series was available. Most (17/24) of the tags were programmed to release from the skate if depths greater than 1700 m were recorded; none of them detached because of this feature. The tags were not programmed to pop-off if no vertical movement was detected for a certain number of days because there was no prior knowledge on vertical movements for this species.

The absence of discernible vertical movement was not automatically considered as an indicator of mortality, as the low depth resolution could mask depth-restricted movements. Tag mobility and other accelerometry-derived metrics were used as secondary indicators of mortality and to identify any changes in activity during the deployment that could be attributed to mortality. Overall mobility of tags was determined through Hidden Markov models (HMM) using the tag mobility metric calculated every 2 h. The HMMs defined two states: ‘mobile’ and ‘non-mobile’, serving as proxies for survival. Model parameters were estimated using the Baum–Welch algorithm, assuming a normal distribution of responses.

Increases in tag mobility were not necessarily caused by skate activity. The tags were attached to the skates’ tail by a short monofilament line and are slightly positively buoyant, so they stood vertically if no force was applied to them. If the skate moved, the tag was pulled by its tether, trailing behind the animal and mobility increased. However, the tag could also be pushed down by currents or animals such as crabs [37,45]. Currents of 1 knot (0.5 m s^−1^) can exert a vertical acceleration component, Az, of −0.5 g and push the tag down around 45°, simulating slow skate movement [45]. Deep-sea bottom currents are extremely complex and will vary over tidal and seasonal time scales [47]. Large-scale modelling indicates a combination of geostrophic and tidal currents can reach close to 0.5 m s^−1^ in areas East of Heard Island [48]. Therefore, exogenous factors such as tides could cause periodic peaks. Lomb−Scargle periodograms were computed with both tag mobility and depth to identify any diel patterns or other periodicities in the time series.

While an increase in tag mobility can indicate activity from the skate or any exogenous source, a tag experiencing only gravitational acceleration must be on a skate that is not active [45]. Based on the recorded tag mobility value, each 2 h period (or 1 h, for pilot tags 85 and 101) was categorized as being ‘still’ (tag mobility <9) or ‘not still’ (tag mobility >9). This mobility cut-off value corresponds to no forces other than gravitational acceleration (−1 g) acting on the tag for most of the 2 h period [44]. We summarized for each skate the percentage of time (in 2 h increments) the tag was still, the number of times it was still for more than 24 h and the amount of time the tag was tilted (*>*−0.75 g). All tags had a non-zero baseline value for tag mobility, which accounts for sensor error and/or angled resting position.

Overall mean tag mobility, percentage of time still, number of hours the tag was tilted and vertical mobility, in the context of sea floor topography and currents, were taken into account to determine mortality. Non-metric multidimensional scaling (NMDS) condensed the inputs from the former four parameters to assess whether the activity metrics and type of detachment aligned with the absence of discernible vertical movement in the determination of post-release survival. We used the function meta-MDS in the R package *vegan* to perform NMDS using Bray–Curtis dissimilarity. Once a post-release survival rate was estimated for *B. irrasa* caught 1200−1600 m, we used the function binom.confit in the R package *binom* to obtain a 95% confidence interval. To identify factors influencing the post-release survival of skates caught on deep-set longlines, we applied a binomial generalized linear model (GLM) with a logit link function. The model included capture depth, total skate length, sex, time out of water and maximum soak time as predictors, with skate survival treated as a binary response variable. Capture area was excluded as a predictor because it confounded the results, likely due to strong interactions with the depth variable.

## 3. Results

### (a) Tagging statistics

**Figure 1.**
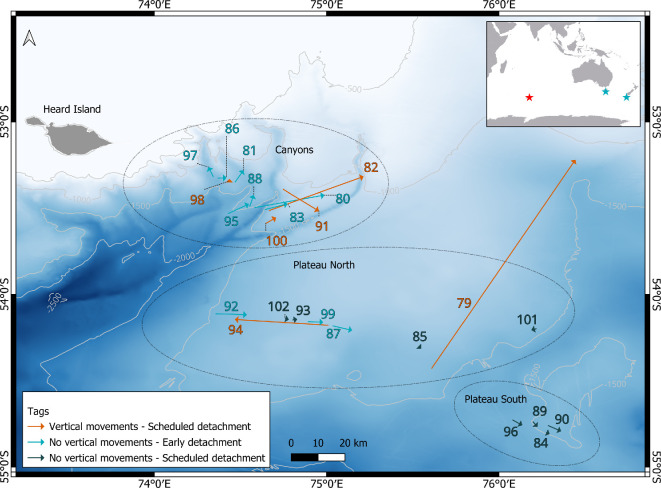
Deployment and estimated pop-up location for 24 tags deployed on Kerguelen sandpaper skates, *Bathyraja irrasa*, captured by demersal longline. Each arrow goes from the deployment location to the estimated pop-up location. The red star indicates Heard Island. The blue stars indicate Tasmania, Australia and Stewart Island, New Zealand, where tags 98 and 93 were retrieved, respectively. Isobaths are in metres.

Over 300 000 hooks were observed during 44 different hauls over the course of 1 month to tag 23 skates in releasable condition and one control skate (107–130 cm total length) (electronic supplementary material, S2). A total of 519 skates were caught in those 44 hauls, 387 of which were released. All deployed tags successfully returned to the surface after detachment, transmitting on average 80% of the recorded data to the ARGOS system. Remarkably, two tags were retrieved by members of the public after drifting significant distances: tag 98 was found after drifting over 5000 km for 10 months to Tasmania, Australia, while tag 93 (the control tag) was retrieved after drifting over 6000 km for 12 months to Stewart Island, New Zealand. A 50/50 target ratio of male to female tagged skates occurred organically. Mature skates caught were observed to be lethargic on the vessel, displaying pale pink gills rather than the ideal red coloration. None of the skates were observed actively swimming downward upon release and none were subject to observed bird predation. The skates were caught and released in three general areas: (i) the Canyons, on the slopes of Heard Island where depths range 600−1900 m and bathymetric slopes can reach 40° angles, Plateau North, a region of flatter topography and depths ranging 1200−1500 m, (ii) Plateau South, which is similarly flat but in slightly deeper (1500−1600 m) waters of the Kerguelen Plateau and in close proximity to much deeper waters (figure 1). A total of 14 tags popped up on schedule; 12, including the control tag, after 30 days, and the 2 pilot tags after 5 days. A total of nine tags popped up early (13–27 days at liberty). These early detachments could be due to the monofilament coming free of a consumed carcass or tag attachment failure. Finally, one tag (99) surfaced on day 30 with its pin intact, a few hours before the burn release sequence was due to be activated to detach the tag.

### (b) Post-release survival

Based on the lack of vertical movement along with evidence from mobility data (see below), we identified 6 survivors, 16 mortalities (including the control tag) and 2 likely mortalities or severe sublethal effects (86 and 97). The six survivors had vertical movements greater than the depth sensor error (±13−18 m). Five of the six underwent extensive repeated ascents and descents over several hundred metres, consistent with active movements of live skate behaviour (electronic supplementary material, S3). There were no significant changes in temperature, which could indicate the tags had been ingested by an endothermic predator. The sixth tag (94) was released in an area with a very gentle slope (*<*1°) and only recorded downward movement; however, it was still for less than 1% of deployment time and travelled over 36 km between release and pop up, in roughly the opposite direction to all other tags. Although water currents may influence skate movement during post-release descent from the surface, advect a skate carcass on the bottom or carry tags as they float to the surface after detachment, it is unlikely that such extensive movements were due only to currents.

**Figure 2.**
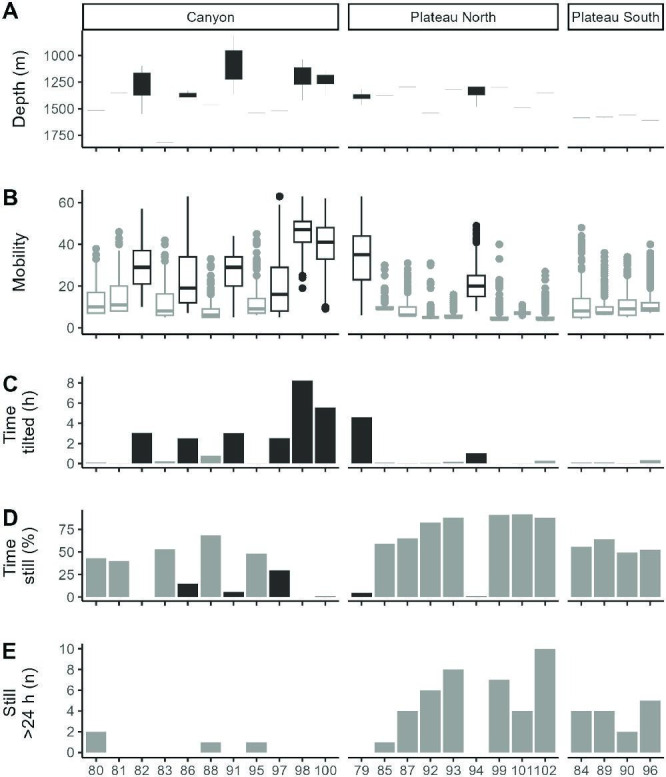
(A) Distribution of depth values for 24 Kerguelen sandpaper skates, *Bathyraja irrasa*, captured by demersal longline. (B) Distribution of tag mobility values. (C) Number of hours tags experienced at least −0.75 g, based on the ‘upright’ metric. (D) Percentage of time (in 2 h increments) where tag mobility <9. (E) Number of periods with tag mobility <9 (‘still’) for over 24 h. ‘Mobile’ tags, as identified by the HMM are black, ‘non-mobile’ tags are grey. Skate 93 is the dead control tag.

A total of 18 tags (including the control tag) recorded no vertical movements greater than sensor error (±13−18 m) during the bottom phase, although one (86) shifted down by one bin depth (35 m) once (figure 2). We further analysed activity metrics to verify that a lack of detectable vertical movements was correlated with a lack of activity and therefore strongly suggested mortality.

The HMMs based on tag mobility suggested that all tags stayed in one state for the full length of their deployment. The model classified eight tags as ‘mobile’ and 16 tags (including the control tag deployed on the euthanized skate) as ‘non-mobile’ (figure 2). The average tag mobility metric of the control tag was very low—it was still 88% of the time—but not zero. Small peaks in tag mobility were recorded throughout its 30 days on the bottom. The six skates with extensive vertical movements, along with two other skates, were classified as ‘mobile’.

The remaining two ‘mobile’ tags (86 and 97) did not record discernible vertical movements during the benthic phase of the deployment beyond one downward shift for 86 and detached early (electronic supplementary material, S2). ‘Non-mobile’ tags had a bottom-heavy distribution of tag mobility. ‘Mobile’ tags had a symmetric or top-heavy distribution of tag mobility, except 86 and 97, which were bottom heavy (figure 2). These two tags were tilted (experienced over −0.75 g) for at least 2 h of their deployment, similarly to skates with vertical movement. Periodograms demonstrate tag mobility peaks for these two skates followed an approximately 24 h periodicity (respectively, period = 24.7 h, PNmax = 0.09, *p* = 0.02 ; period = 24.3 h, PNmax = 0.1, *p* = 0.03). This periodicity was not linked to any vertical movements since there were almost none detected for these tags. The 24 h periodicity could be linked to the tidal cycle, tag mobility values were higher on days around spring tides. For the control tag (93), 87% of 1 Hz tag mobility values higher than nine occurred within 2 days of spring tides. Although skates 86 and 97 had some peaks in tag mobility on par with other ‘mobile’ tags, tag mobility was more often low (*<*20).

While a non-still tag can indicate activity from the skate or any exogenous source, a still vertical tag strongly indicates that the skate was not active. All ‘non-mobile’ tags were still at least 40% (mean % stillness) of the time (figure 2). Additionally, all ‘non-mobile’ tags on the plateau, had at least one continuous period of stillness of over 40 h. All tags in the canyon were still less often than tags on the plateau. Archived data reveal that skate 98 remained stationary for nearly an entire day longer during the first half of its deployment (53 h) compared with the second half (30 h). The six ‘mobile’ tags that had extensive vertical movements were still less than 6% of the time; their vertical migrations indicate they were actively swimming during the non-still periods. The two other ‘mobile’ tags, which had no extensive vertical movements, 86 and 97, were still, respectively, 15 and 30% of the time. Despite having strong periodic peaks in mobility during a few days, their proportion of time still was three to five times greater than any of the other six ‘mobile’ tags.

**Figure 3.**
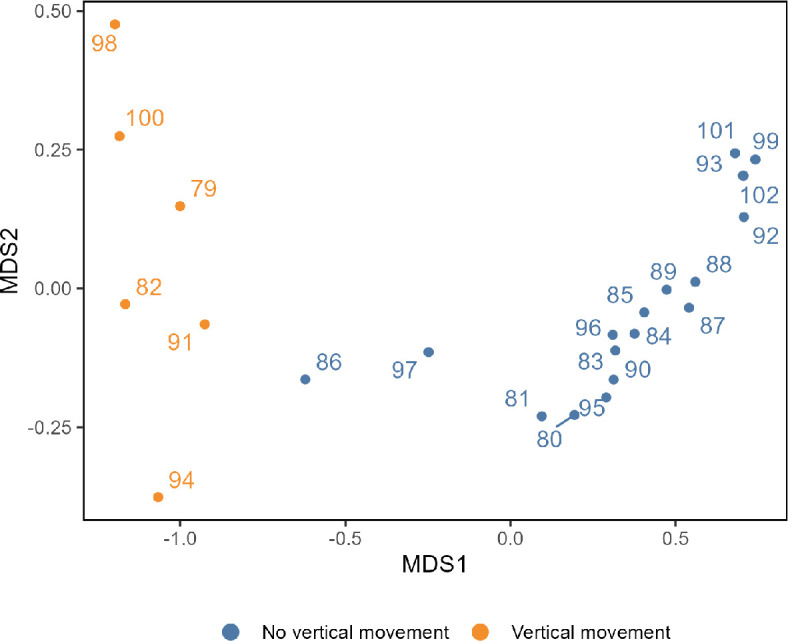
Non-metric multidimensional scaling (NMDS) analysis using Bray–Curtis dissimilarity on the total deployment mobility mean, number of hours the tag was not upright, percentage of time still (tag mobility <9 in 2 h increments) and detachment type (scheduled detachment or early detachment). The colours of the data points correspond to the level of vertical movement detected (see key). Skate 93 is the dead control tag.

An NMDS plot was performed to identify distinctive patterns in activity that could be attributed to live and dead skates (figure 3). The analysis was conducted in two dimensions yielding a stress value of 0.009, indicating a good fit of the ordination. Two clusters of patterns in activity converged with vertical mobility confirming most skates with no detectable vertical movements were not active. We identify six surviving skates clearly distinct from the 16 inactive skates which are estimated mortalities. Tag 86 and, to a lesser extent, 97 are not clearly part of one cluster or another but had characteristics from both.

While the sample size is relatively small for a survival study, considering 86 and 97 as mortalities, confidence intervals indicate post-release survival for *B. irrasa >*1056 mm caught at 1250−1550 m is below 50% and could be as low as 13% (electronic supplementary material, S4). Deploying more tags could provide a more precise range. Assuming the observed probability of survival (26%) (95% CI 13−46%), a power analysis indicates that a deployment of 105 tags would be required to reduce the width of the confidence interval by half (i.e. 18−35%). Reducing the confidence interval further would require a prohibitively large sample size (e.g. 1000 deployments to reduce the width of the CI to 5%).

### (c) Factors in post-release survival

**Figure 4.**
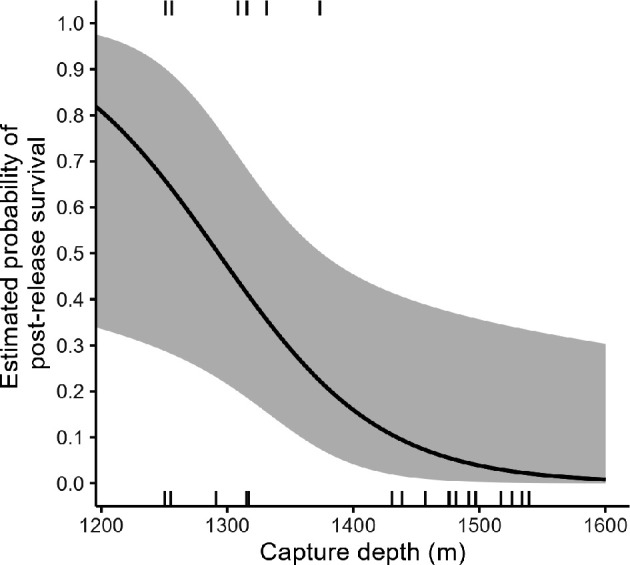
Predicted 5th and 95th percentile values around the post-release survival estimate of *B. irrasa* caught in the Heard Island and McDonald Island Patagonian toothfish demersal longline fishery for a given depth. The 24 skates’ capture depths are plotted in the top (survival) and bottom (mortality) margins.

If skates 86 and 97 were indeed mortalities, all the skates caught deeper than 1450 m died, while all surviving skates were caught shallower than 1350 m (figure 4). The logit regression model indicated capture depth was a significant factor in survival (*p* = 0.02); sex, total length, time out of water, capture area and soak time were not. The odds of survival increased fivefold (odds ratio = 95% CI: 1.5−31.2) for every 100 m shallower a skate was caught.

## 4. Discussion

### (a) Post-release survival rate

This study estimated for the first time the post-release survival of deep-sea skates caught on demersal longline using PSATs. We found that *B. irrasa* over 1056 mm in total length caught in the HIMI Patagonian toothfish longline fishery 1250−1550 m and released at sea in good condition had a low survival rate (26%, 6/23), which was affected by capture depth. Even for survivors and potential survivors, levels of activity suggest some skates had sublethal effects that could, in turn, affect general fitness and may cause delayed onset mortality. The survival at 30 days is therefore possibly an overestimation of post-release survival for this size class at this depth range. For skate 98, the difference in still time between the first 15 days of deployment and the next 15 days indicates even surviving active skates may require increased resting time in the first 2 weeks after release. For skates 86 and 97, while tag mobility was sometimes high and the tag was pushed down as much as some surviving skates, they were still relatively often, showed no or limited detectable vertical movement and the tags detached early. There are three possible interpretations for these skates’ outcome: (i) they survived but were less active than other surviving skates, moving in a restricted depth range of less than 40 m and resting for longer periods between bouts of activity, then the tag attachment failed, (ii) they survived for a limited amount of days, then died after failing to recover; the tag being freed by lice or other scavengers, or (iii) they died quickly after release and all the increases in tag mobility were due to external forces, whether from currents, predators or scavengers. Independent of which scenario, the data indicates skates 86 and 97 were far less active than other surviving skates and therefore would have sublethal effects that could compromise feeding and long-term fitness if they had survived the length of the tag deployment.

The survival rate of *B. irrasa* from this study is lower than that of other skate species caught in deep-set longlines in the Southern Ocean. While comparisons between studies with different methods should be done with caution, stark differences in survival estimates could indicate inter-species differences. Softnose skates *Bathyraja* spp. may have a lesser resistance to injury from longline capture compared with hardnose skates *Raja* spp. [32,33,49]. Based on mark-recapture data, Whiteleg skate, *Amblyraja taaf* caught in the neighbouring French Crozet Island longline toothfish fishery had a post-release survival rate over 92% [32]. Considerably lower recapture rates were noted for *Bathyraja* spp. in the same study around Kerguelen Island, indicating a marked difference in post-release survival rates and/or population sizes between the species. *Raja* sp. skates captured at 1300−1500 m depth in South Georgia had an estimated 46% survival rate based on 12 h tank trials with skates in good and bad condition [33]. *B. irrasa* are larger than most skates assessed for post-release survival in previous studies [33,50]. In other elasmobranch species, the chances of survival after capture by longline have either increased [32,51] or decreased [52] with increasing size. During this study, while most of the smaller juvenile *B. irrasa* hauled were tonic, with red gills and sometimes curling their tail up like a scorpion, only a few of the mature larger skates were. Most were limp, with the only signs of life being pink gills and spiracle or gills movements. Although this study did not show any effect of size on survival, sample size was relatively small and only skates over 1056 mm in length could be tagged. Therefore, we cannot draw definitive conclusions about the survival rate of smaller individuals, especially because these are usually caught in shallower waters (*<*1000 m). If there is a negative effect of smaller size it would be competing with the positive effect of shallower capture depths for this subset of the population.

Survival after release is influenced by the effects of capture during every step of the fishing process, from hooking to hauling, handling and method of release [28,29]. Fishing at shallower depths could improve survival of *B. irrasa*. While *Raja* sp. caught in South Georgia had decreased likelihood of survival with increasing capture depth, *Amblyraja taaf* caught in the Crozet Islands fishery had a stronger likelihood of survival at mid-depths (1200−1500 m) and the lowest likelihood at depths shallower than 1200 m [32,33]. Faure *et al.* [32] suggested this reversal could be due to increased hauling speed for lines set shallower. In the present study, the 300 m range in capture depths was relatively small in the context of this fishery, but enough to suggest a negative effect of capture depth on survival. No skates caught shallower than 1200 m were included in this study so any reverse effect of shallower depths could not be observed. Although sex, total length, time out of water and soak time did not significantly affect survival in this study, the relatively small sample size may be masking their effects [53,54]. Other aspects of fishing technique may also factor into survival: type of hooks used [55], method of release [56] or hauling speed [32]. The use of chutes during release, for example, in bunker-style hauling rooms, could minimize impact with the sea surface and in moon pool vessels could protect skates from getting tangled and hooked again in the ascending line. Tank trials in South Georgia may have overestimated post-release survival as skates were not returned to the sea where they may encounter predators [28,57]. For deep-sea skates in the Southern Ocean, micropredators may be just as dangerous as large predators such as sleeper sharks [58,59]. Many species of amphipods and isopods form part of a macrobenthic community which scavenges on food falls, but some crustacean species with good swimming abilities are also known to prey on live or moribund benthic fish [60,61]. Scavenging and micropredatory crustaceans, or sea lice, may opportunistically prey on weakened or moribund skates, thereby reducing chances of recovery after capture. Sea lice concentration and distribution could therefore be a factor in the survival of skates [62,63]. Skates may survive the initial stress of capture but be too weak to continually evade predators, thereby further reducing their chances of survival. Mounting cameras recording for 48 h after release before popping up to the surface, with as much of the skate body in view could reveal whether—and how quickly—crustaceans make contact with skates upon return to the sea floor.

Passive descents and lack of change in mobility and depth patterns indicate mortality occurred within a few hours after release. This aligns with the South Georgia tank experiments in which 44% of the skates were observed dead within 12 h and 14% were in a severe state, unlikely to survive [33]. This rapid onset of mortality indicates skates are moribund on the vessel. However, it is extremely difficult to predict the outcome for skates with no severe injuries based only on physical condition at time of hauling. No skates released in this study had observable severe injuries, yet the survival rate was low. This suggests that severe metabolic stress from exhaustion, which is not necessarily externally visible, or internal injuries, could play a significant role. While severe injuries may be strong indicators of increased mortality risk, the absence of severe injuries and the presence of pink gills can in no way predict survival. However, the prognostic value of moderate to severe injuries remains unverified; tagging skates with varying injury severities would provide insights into how injuries influence survival outcomes. The proportion of skates with mild to severe injuries depending on capture depth, soak time, sex and total length would also be needed to properly estimate total fishing mortality. These data are currently not available. In the meantime, unless the condition of skates on landing can be notably improved (red gills, tonic state) monitoring tools complimentary to PSATs should be developed to better understand what factors affect the post-release survival of deep-set longline-caught skates. Blood stress markers may help in identifying such factors.

### (b) MiniPAT tags as indicators of survival for deep-sea benthic fish

The difficulty in assessing the fate of low-activity skates highlights the limits of PSAT technology in the determination of survival. Assessing the survival of a data-poor, remote, deep-sea demersal species was challenging but miniPATs provided an estimation of activity that was used as a proxy for survival in an extreme environment. However, these PSATs could not unequivocally distinguish an extremely sluggish, seldom active animal from a dead one. If a fish is resting most of the time and has very limited vertical movements when they are active it will appear as an evocative flat line both on depth and mobility records, especially in extreme depths where sensor error carries a range of ±1% of the recorded depth. Considering the topography of the release area is essential, because it is easier to identify an active skate from depth data in an area with a high slope as opposed to a big plateau or gentle slope. Skate 94 may have been moving within a 35 m depth range in the first 2 weeks, as suggested by relatively high mobility values, but it is hard to confirm because the slope on the plateau is so gentle, providing a very large area within restricted depth ranges (15–25 km wide band). On the other hand, if skates 86 or 97 were actively swimming during most non-still times, as is the case for the six skates that showed vertical movements, they would have had to be moving strictly within a 1−6 km wide band in the canyon area where slopes can be as high as 40°. This would indicate a strong attachment to a specific depth, which has not been observed in other skate telemetry studies or in active skates in this study [64–67]. Some shallower species have displayed such reduced movements, in the 2 weeks following tagging [67]; and deep-sea Arctic skates tagged in the Cumberland sound with a range of vertical activity similar to those of active skates in this study, had restricted vertical movement for up to 7 days after tagging [66]. All these skates undertook large vertical migrations during or after that initial recovery period, unlike skates 86 and 97, which remained within a 35−40 m depth range. For skate 94, the limited vertical movement observed during the first 2 weeks may be due to reduced activity as the skate recovered, rather than solely being caused by low depth resolution and a flat sea floor. Further studies are needed to confirm whether some *B. irrasa* are extremely active while others are not or simply take longer to recover from capture. Even if non-active skates are not dead, such low activity for a prolonged period would affect food intake and therefore general fitness and long-term survival. Stationary times longer than 2 weeks, as may be the case for skate 94, have not been reported previously for other skate species [64,66,67]. Short high-resolution deployments with other monitoring tools such as heart monitors [68] or cameras [69] could improve our understanding of the physiological state of inactive skates; to distinguish normally sluggish skates from dead skates and recovering skates. The high-activity (HA) data transmitted by the miniPAT was not usable to determine survival or activity in this study. The HA events count may be appropriate for animals that move through bursts of movements but the count was too often very high for *B. irrasa*. As this metric is based on 1 Hz mobility values above the 2 h mobility mean, very small increases in mobility in an otherwise slow-moving or dead animal were recorded as HA events.

Early detachment appears to be a strong indicator of mortality due to the fact that all the surviving skates retained their tags for 30 days. We attribute these premature detachments to scavenging sea lice. While they preferentially consume soft tissues, they eventually attack tougher tissues like the ones connecting cartilaginous vertebrae, which presumably would reduce the integrity of a skate tail until the monofilament tag tether is liberated [70]. The fact that all non-mobile tags in the Canyons detached early while only a portion of those on the plateau did is possibly due to a higher concentration of lice in the Canyons region ([71]). The control euthanized skate retained its tag for the full deployment, indicating scavengers can take more than 30 days to liberate a tag with a tail attachment. A patchy sea lice distribution on the plateau could explain why some of the tags on dead skates did not detach early. Longer deployments could help better understand the role of scavengers in the early detachment of tags on dead skates. Using more cost-effective PSATs, such as sPATs that record depth but not acceleration, could suffice to infer mortality for *B. irrasa* while enabling the tagging of a larger number of animals.

This study provides a first estimate of survival for skates in the HIMI Patagonian toothfish fishery, which can inform bycatch stock assessments. The high mortality rate of *B. irrasa* over 1056 mm calls for mitigation of the effects of fisheries interaction, through catch avoidance or increase of survival. The effect of depth suggests fishing in shallower waters could improve survival. CCAMLR conservation measure 32–18, which indicates sharks should be released alive, is clearly insufficient for ensuring the survival of incidental bycatch in deep-set demersal longline fisheries as releasing animals alive does not ensure their survival after release [26]. Further research is needed to identify predictive cues other than physical condition and to better understand the factors affecting survival, such as size, fishing technique, handling and release methods. Improving the at-vessel condition of mature skates would be a first marker of success.

## Data Availability

Data and code : Institute for Marine and Antarctic Studies [46]. Supplementary material is available online [72].
